# Analysis of the reduction processes at the bottom of Lake Meirama: a singular case of lake formation

**DOI:** 10.1007/s10661-023-11604-z

**Published:** 2023-07-27

**Authors:** Ricardo Juncosa, Jorge Delgado, José Luis Cereijo, Andrea Muñoz

**Affiliations:** Civil Engineering School, University of Coruña, Campus de Elviña, 15071 La Coruña, Spain

**Keywords:** Reduction processes, Lakes formation, Mine waters, Methanization, Anoxia

## Abstract

The formation of natural lakes is a process that takes place over thousands of years, although the volumetric formation depends on hydrological and climatological phenomena, reaching a stationary hydraulic regime, the evolution of hydrochemistry is more complex and obeys not only phenomena of stoichiometry and chemical kinetics but also diffusion processes. Depending on the depth of the lakes, the anoxization process originating from the bottom is the first phase of the lake’s methanogenesis. For this, the course of many thousands of years is necessary, so the studies carried out in the lakes are limited to the current knowledge of the state in which they are, without being able to have real information in this process of methanogenesis. There are no data available on the generation process of a natural lake in its primary stages. In this case, taking advantage of the rehabilitation of the old open-pit mining of Meirama (Northwest Spain), consisting of the controlled flooding of the hole by groundwater, by stopping the perimeter pumping, and the derivation of the nearby streams, whose contribution was the majority with respect to the subterranean contribution, there has been the opportunity to physically and chemically monitor the complete filling of the said hole. The present study focuses on the analysis of the evolution of the different processes initiated in the methanogenesis of the lake bottom identified in the well-known Redox ladder: obtaining oxygen from the reduction of nitrogenous compounds and metallic oxides, from the reduction of the sulfate and the generation of methane from carbon compounds, the latter phase without reaching. Although the methanization process is very slow, it has had the opportunity to know the formation of a lake at its origin, from the hydrochemical point of view. It has been possible to verify that the methanization processes at the bottom, given the anoxia conditions, are in a very primitive phase with the reduction of nitrate and nitrite to ammonium and beginning a reduction of metal oxides and sulfate.

## Introduction

**Fig. 1:**
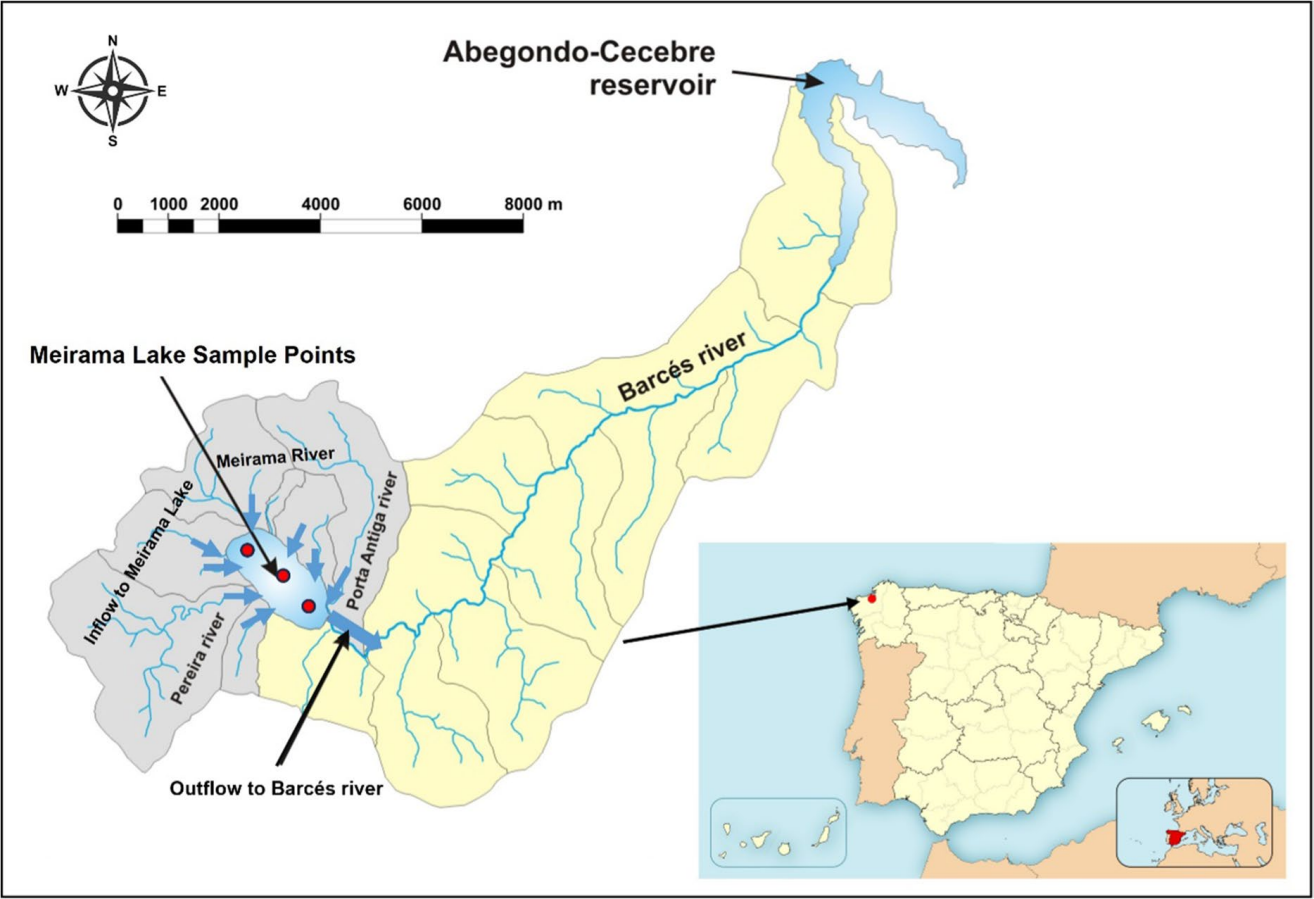
Location of the Meirama pit lake (own elaboration). Grey zone: tributary basins to the open-pit

The Meirama mine (northwestern Spain) was a brown coal mine that supplied a thermal power station for electricity generation (Fig. 1). The mineral was exploited between 1980 and March 2008. Lignite mining generated a 2.2-km-long and 1-km-wide water-filled hole sized approximately 146 hm^3^. The maximum depth measured in the lake was 205 m (Juncosa et al., 2018). The lake is the first mining hole in the world rehabilitated as a water supply reservoir for a large population (Hrdinka, 2005; McCullough et al., 2020; McCullough & Schultze, 2018a, 2018b; McCullough & van Etten, 2011; Stephenson & Castendyk, 2019; Vandenberg et al., 2011; Vandenberg & Litke, 2017; World Bank, 2005). More specifically, this lake supplies the city of La Coruña and its metropolitan area (Fleischhammel & Menéndez-Lolo, 2010) as an auxiliary reservoir to the Abegondo-cecebre reservoir, located 13 km downstream of the mining lake.

The pit had a system of perimeter wells that pumped water during ore extraction (4 hm^3^ per year), in addition to a pump at the bottom of the mine. In March 2008, when the mining ceased, perimeter pumping as well as pumping at the bottom of the mine stopped, and the mine began filling with groundwater. At the end of September 2008, the surrounding streams (Pereira river, Meiram river and Porta Antiga river) began to be diverted into the pit to accelerate filling and to improve water quality (Juncosa et al., 2008; Schultze et al., 2002; Schultze, Geller, et al., 2011a; Schultze, Pokrandt, et al., 2011b). To control the hydrochemical quality of the filling, the evolution of the chemical quality of the waters of the hole has been systematically monitored (Delgado et al., 2013; Søndergaard et al., 2018). For this reason, during filling and post-filling, the filling process of the mining pit was monitored (Gammons et al., 2009; Zhao et al., 2010).

The formation of natural lakes is a complex process that lasts for long periods of time. At our time scale, it is only possible to analyze and study the verification of the generation of certain physical and chemical processes at present. In this case, it has had the opportunity to study the formation of a lake from its origins and corroborate the different physical and chemical processes that have been developing.

This article analyzes the anoxification process that occurs at the bottom of the pit together with the evolution of some major components. The parameters and compounds chosen are pH, dissolved oxygen, nitrate, nitrite, ammonium, total nitrogen, sulfate, iron, manganese, and dissolved inorganic carbon (DIC).

The objective of the study is to analyze the evolution of these parameters and compounds at the bottom of the open-pit lake over 3 years (2016–2019), once the open-pit was filled.

## Materials and methods

**Fig. 2:**
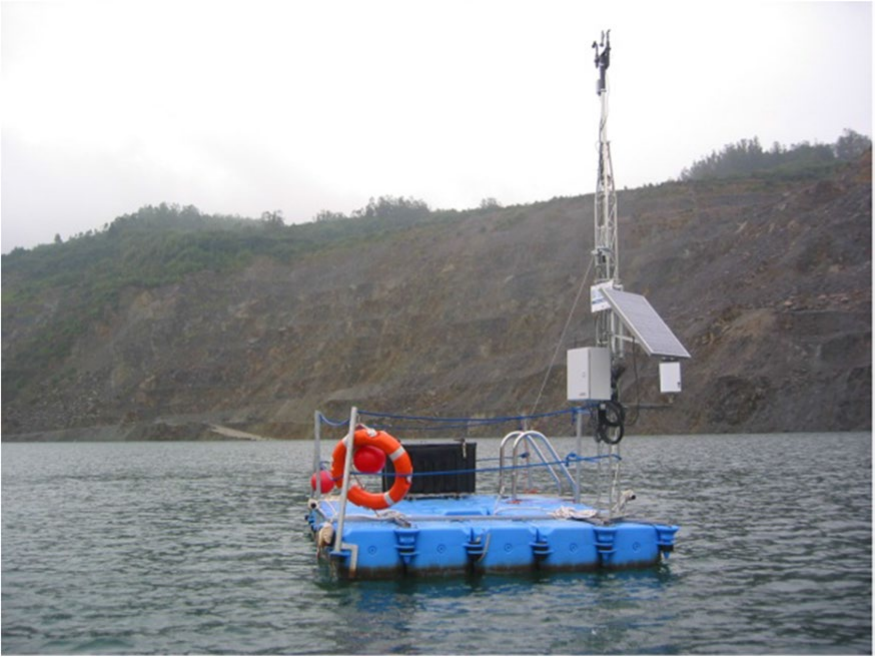
Sampling platform

The systematic sampling consisted of monthly samplings of vertical profiles in three points of the lake until December 2013. When the profiles were the same, regardless of the sampling point (stratification of the lake), a single monthly sampling at the deepest point was performed using a floating platform anchored to the bottom that rose as the hole filled (Fig. 2). From March 2008 to June 2010, the lake was sampled every 5 m. From July 2010 to February 2011, samples were taken every 5 m up to 40 m in depth, and thereafter, every 10 m from the bottom. From March 2011 to December 2012, samples were collected every 10 m from the first 30 m of depth and every 30 m from 30 m of depth, that is, 60, 90, 120, and consecutive depths.

From January 2013, the sampling depth distribution was as follows: surface, every 5 m for the first 20 m of depth, and every 10 m thereafter, that is, 30, 40, 50, 60, and consecutive depths. Samples were collected monthly.

Sampling was performed according to the conventional techniques described in ISO 5667-1:2006 (Water quality, 2006), ISO 5667-2:1991 (Water quality, 1991), and ISO 5667-3:2003 Water quality, 2003). The pH, dissolved oxygen concentration, and oxidation-reduction potential (ORP), among other parameters, were determined at the same sampling point. The remaining parameters were measured in the laboratory according to specific techniques and procedures (Apha, 1998; Appelo & Postma, 1992; Hounslow, 1995).

A YSI 556 MPS multiparametric probe was used to measure the surface water (Yellow Springs Instruments, 2006). The parameters measured with this equipment were the pH (combined glass electrode), ORP (Pt electrode), and dissolved oxygen (“polarographic steady state” membrane sensor), among other parameters. In addition to these parameters, the YSI 6600 V2 probe was used to measure the vertical profiles in the lake up to 220 m of depth. With the YSI 6600 probe, vertical profiles were recorded every 2 m, stabilizing the probe at each depth for 2 min.

For deep sampling, a Kemmerer bottle was initially used and then replaced when reaching higher lake levels by an automatic raising and lowering system with a Carousel sampler (SBE 55 ECO water sampler), with 6 bottles with a 4-L capacity each and with a magnetic closure system; this setup collected up to 6 water samples at different depths in each dive.

## Results and discussion

Initially, when the mine pit was filled, the water level rose rapidly (Hernández et al., 2012), subsequently slowing as the flood area increased. The reactive capacity of sulfides causes their oxidation, increasing dissolved solids as the water becomes acidic (Delgado et al., 2008, 2014; Schultze, 2012). As a result, the water contains high concentrations of metals and sulfates and an acid pH (Bylak et al., 2019).

**Fig. 3:**
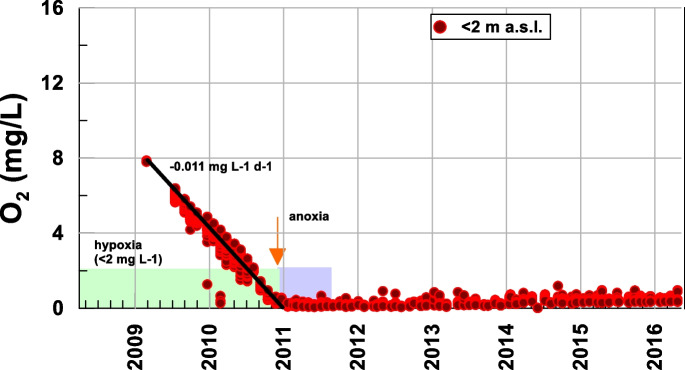
Temporal evolution of the dissolved oxygen at the bottom of the lake

The mining lake is a complex system in which different factors affect the water quality evolution (Wolkersdofer, 2008). Below, Figs. 3, 6, 7, and 8 show the results for different physicochemical parameters (pH and dissolved oxygen) and some cations (Mn and Fe) measured at the bottom of the open pit. Initially, the pit was filled with groundwater (March–September 2008); for this reason, the water at the bottom matched the water of the initial lake. Subsequently, the waters of nearby rivers were diverted towards the pit, producing a total mixture. As the water level rose, a mixing zone was produced in the top layer, forming a monimolimnion and a chemocline (Delgado et al., 2013).

However, the deepest section remained mostly unchanged until the stored volume began to increase, starting the bottom anoxification. Figure 3 shows the evolution of dissolved oxygen, which qualitatively corroborates the findings of different authors reported in limnological studies conducted in different lakes (Miller et al., 1996; Nürnber, 1995). Thus, the process of the oxygen concentration decay was studied, and it was compared with the redox processes that lead to the methanogenesis of the lake at the maximum depths, where different species are reduced (kalff, 2002).

The concentration of dissolved oxygen in water is a key indicator of its ecological status (Carlson, 1977; Miller et al., 1996; Nürnber, 1995; Rzetala & Jagus, 2012; Wetzel, 1983). Available data show, without a doubt, the gradual consumption of oxygen in deep areas of the lake, with a clear drift towards anoxia (Miller et al., 1996; Nürnber, 1995; Ramstedt et al., 2003). This oxygen consumption is most likely due to the oxidation of metals, with a decay rate of −0.011 mg L-1 d-1 (−3.6·10^−4^ mmol L-1 d-1), reaching anoxic conditions in the mid-2010 and hypoxic conditions in the late 2010 and maintaining these conditions virtually until the end of the filling (Table 1).

**Table 1:** Average rates of change of the different compounds

	2009	2010		2011	2012		2013	2014	2015	2016
pH	4–5	5–3		3–3.5	3.5–4		4–4.5	4.5–5	5–5.2	5.2–5.5
O_2_ (mmol L-1 d-1)	−3.6·10^−4^			0						
NO_3_^−^ (mmol L-1 d-1)	3.22·10^−5^	−2.4·10^−4^	2.4·10^−4^	−9.67·10^−5^			0			
NO_2_^−^ (mmol L-1 d-1)	2.2·10^−5^		−1.48·10^−5^		0					
NH_4_^+^ (mmol L-1 d-1)	5.62·10^−5^		−2·10^−4^	2.2·10^−5^			3.05·10^−5^			
Mn^2+^ (mmol L-1 d-1)	6.36·10^−5^		3.4·10^−4^	1.81·10^−5^						
Fe^2+^ (mmol L-1 d-1)	0			1.6·10^−4^			4.8·10^−4^			
SO_4_^2−^ (mmol L-1 d-1)	4.7·10^−3^	−0.01	0.01	0	−0.008	0.008	0.0012		−0.0013	

**Fig. 4:**
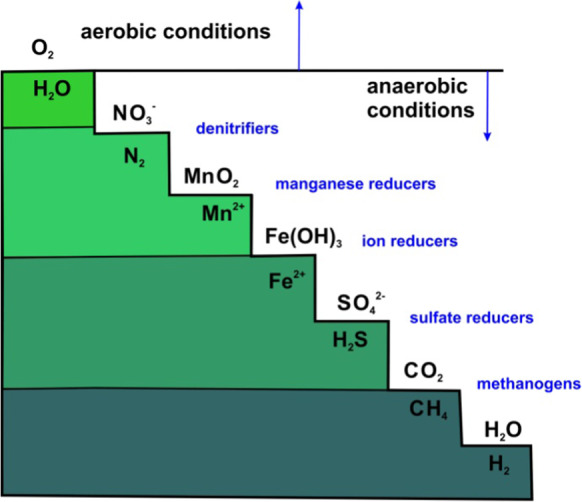
Redox ladder

**Fig. 5:**
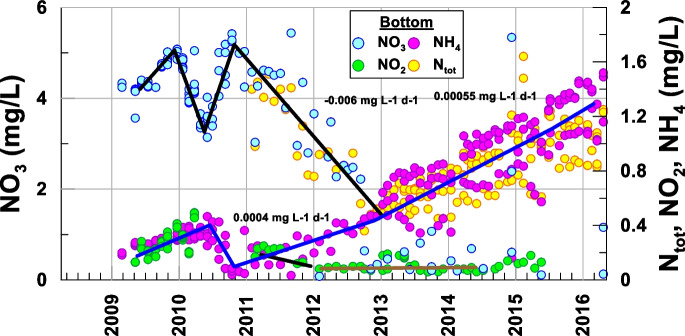
Temporal evolution of nitrogen compounds at the bottom of the lake

Figure 4 shows the redox chain in methanogenesis, processes that have already been observed in deep natural lakes (Lu, 2004) and in flooded mine pits (Bachmann et al., 2001; Denimal et al., 2005; Hamblin et al., 1999; Jonas, 2000; Shevenell, 2000; Shevenell et al., 1999). Depending on the concentration, pH, and Eh of the water at the bottom of the lake, the sequence of the reduction of the different compounds may vary. Thus, initially, the decrease in oxygen, according to Fig. 4, did not substantially affect nitrate or Fe reduction, as shown in Fig. 5. The amount of nitrogen compounds increased in 2009 with the entry of surface waters, supply waters that transported nutrients and whose variability is in line with those identified at the bottom of the lake in the 2009–2011 period, when the lake had not yet reached a determining depth at which the supply waters mixed with groundwater.

**Fig. 6:**
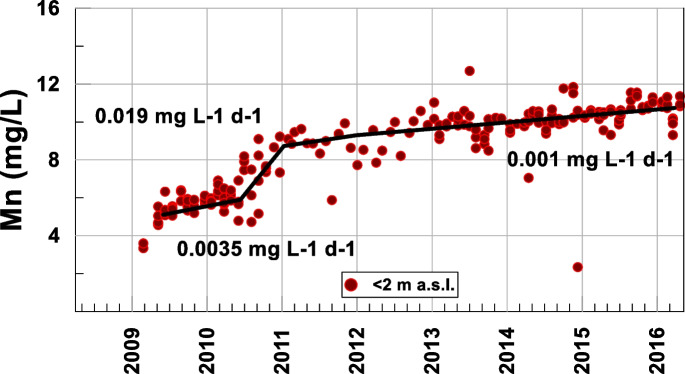
Temporal evolution of Mn at the bottom of the lake

However, manganese began to be reduced since the measured pH (Bylak et al., 2019; Carlson, 1977) and Eh (200–500 mV) conditions favored its reduced form, whose associated species are soluble (Fig. 6), sharply changing the growth rate (0.019 mg L-1 d-1 = 3.4·10^−4^ mmol L-1 d-1) in mid-2010 when hypoxia was reached (Table 1). Similarly, when hypoxic conditions began to occur, nitrogen, which was mainly found in the form of nitrate, began to be reduced to nitrite (Delgado et al., 2014), decreasing its concentration at a rate of −0.006 mg L-1 d-1 (−9.67·10^−5^ mmol L-1 d-1) (Table 1); nitrate, in turn, was reduced to ammonium, with the latter increasing at a rate of 0.0004 mg L-1 d-1 (2.8·10^−5^ mmol L-1 d-1) in the 2011–2013 period (Table 1). However, from 2012, nitrite remained quasi-seasonal (Fig. 5).

**Fig. 7:**
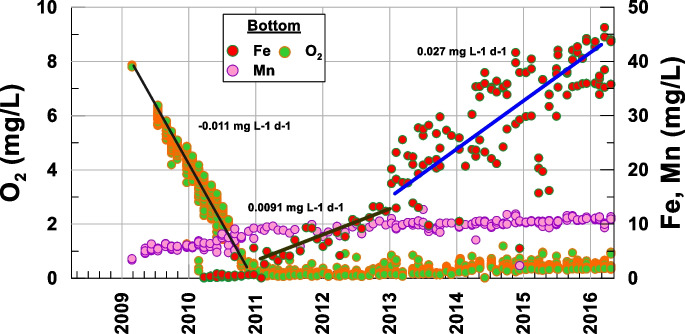
Comparative temporal evolution of oxygen, Fe, and Mn at the bottom of the lake

Figure 7 shows that iron, which initially precipitated in the form of oxides and hydroxides, began to be reduced when anoxic conditions were reached in 2011, competing with the reduction of manganese, which drastically changed the slope of its growth rate (Fig. 6; Table 1) (0.001 mg L-1 d-1).

**Fig. 8:**
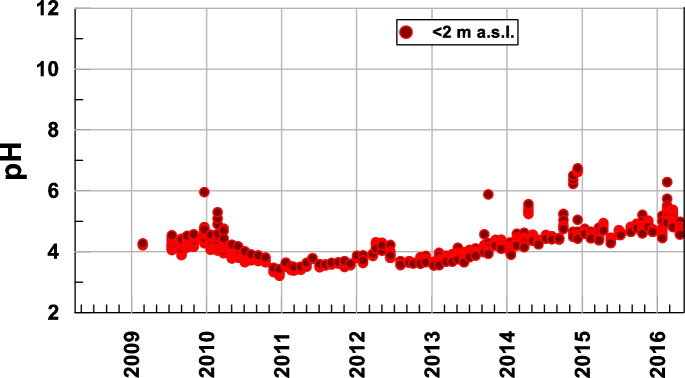
Temporal evolution of the pH at the bottom of the lake

**Fig. 9:**
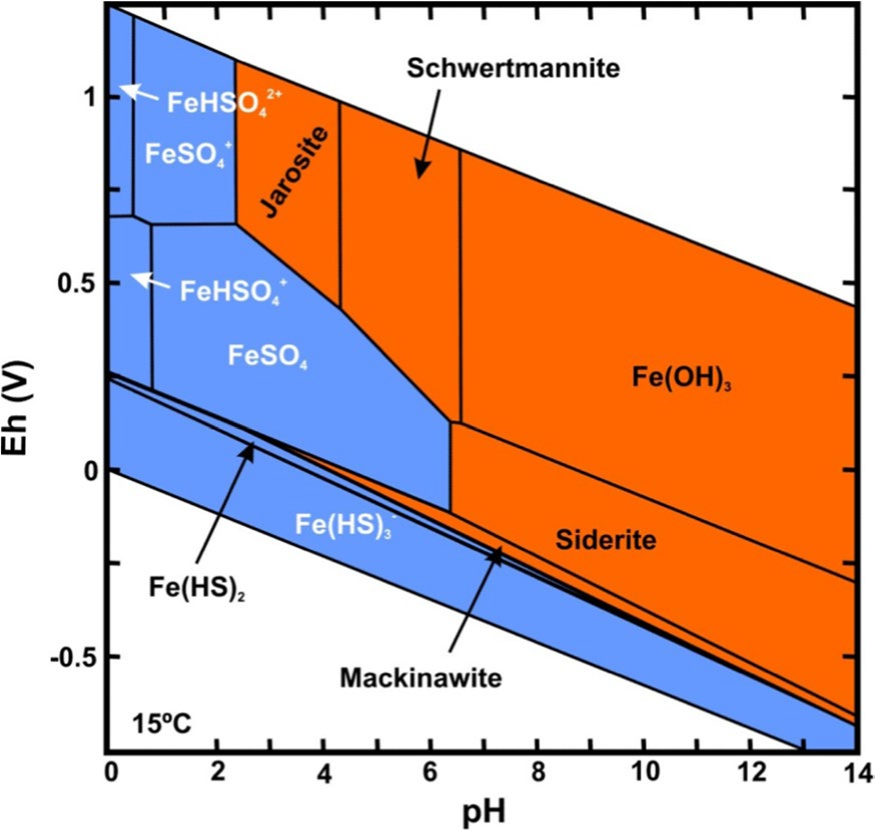
Eh-pH diagram of Fe. Orange areas represent solid phases, and blue areas represent the more stable aqueous phase

**Fig. 10:**
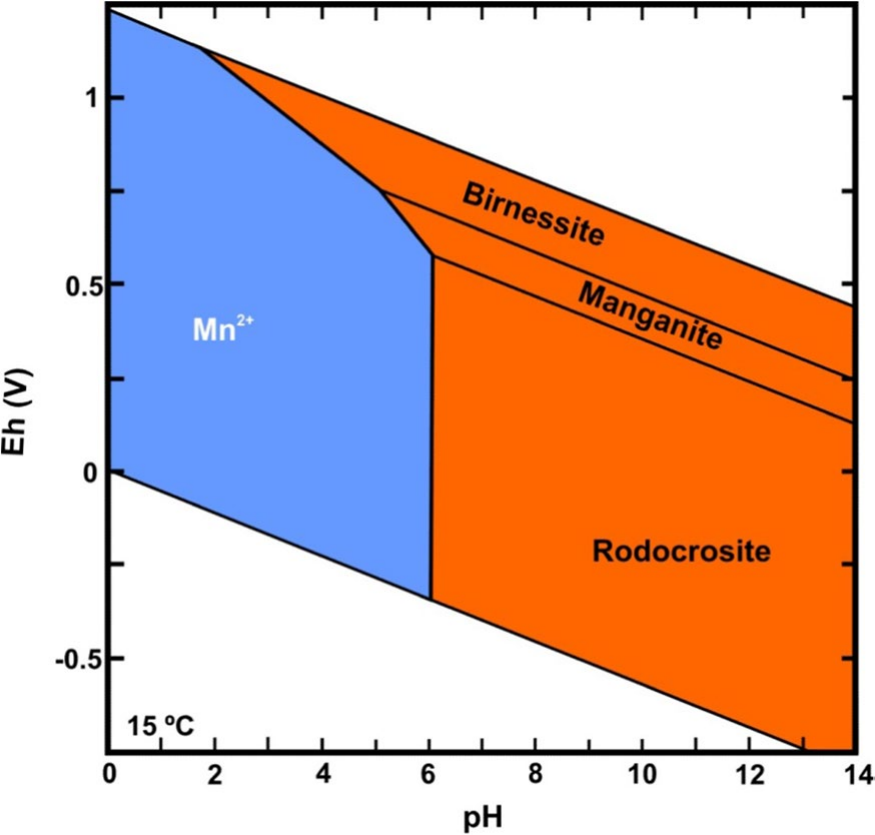
Eh-pH diagram of Mn. Orange areas represent solid phases, and blue areas represent the more stable aqueous phase

While the pH evolution was very minor, with a tendency to increase from 2011, the pH range remained between 3.5 and 5.5, that is, acidic conditions (Fig. 8). Considering that the measured values of Eh ranged from 200 to 500 mV, Figs. 9 and 10 show that Fe and Mn compounds were mainly found in the aqueous phase in reduced form and that some precipitated minerals of the oxides of both metals could be redissolved, as shown by the increase in Mn in solution at a rate of 0.001 mg L-1 d-1 (1.81·10^−5^ mmol L-1 d-1) and that in iron with an initial rate of 0.0091 mg L-1 d-1 (1.63·10^−4^ mmol L-1 d-1) until 2013 and then of 0.027 mg L-1 d-1 (4.83·10^−4^ mmol L-1 d-1) because these compounds are more soluble than Mn compounds, thus matching the change in the ammonium slope (Table 1).

**Fig. 11:**
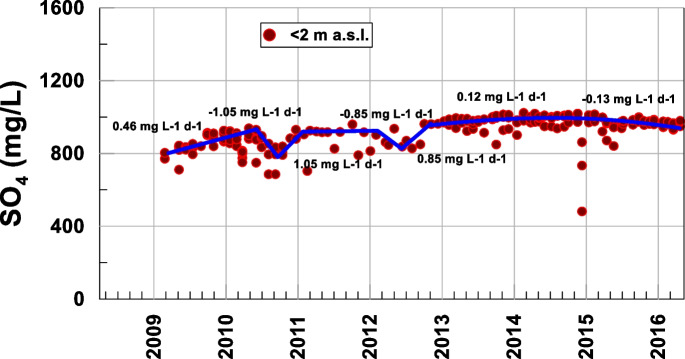
Temporal evolution of sulfate at the bottom of the lake

Figure 4 shows that the next compounds to get the oxygen are sulfate compounds (Fig. 11). The oxygen consumption was most likely related to the decomposition of organic matter, whose contribution came from higher levels. In this layer, sulfate compounds were reduced to sulfides when the oxidant contribution from metal oxides and hydroxides decreased substantially. The concentration of sulfate in aqueous solution generally increased from 2009 to late 2014 (from 0.46 mg L-1 d-1 or 4.7·10^−3^ mmol L-1 d-1 to 0.12 mg L-1 d-1 or 1.2·10^−3^ mmol L-1 d-1) and is now beginning to decrease slightly (−0.13 mg L-1 d-1 or −1.3·10^−3^ mmol L-1 d-1), which suggests that, at the moment, sulfur reduction is not intense, and thus, neither is carbon methanization (Table 1). The time elapsed until now is insufficient for assessing methane, but some overlaps occur in the nitrate, nitrite, Mn, and Fe reduction processes, which have not finished yet.

The increase in sulfate in the 2011–2014 period was related to the solubility of the iron sulfate compounds that were formed (Fig. 9). The increases in both Fe and sulfate are shown in Figs. 7 and 11. Starting in 2015, sulfur began to decrease, which suggests that this element was beginning to be reduced.

Table 1 outlines the rates of change of the different species analyzed in this study. This table highlights different overlaps in the methanization process at the bottom of the lake.

## Conclusions

This article has served to show the different chemical and physical processes that originate at the bottom of a developing lake.

The chemical quality during the process of formation of the Meirama pit lake has been monitored for 8 years. To identify different methanization processes that occur in deep lakes and reservoirs, different components were analyzed in the deep areas of this lake. In the methanization process of Lake Meirama, different redox processes, albeit not yet complete and thus unable to oxidize organic matter, were detected. Those processes included denitrification and ammonification of nitrogen compounds (nitrogen cycle) and Mn and Fe reduction to soluble forms, which increased their concentration in the liquid phase. The reduction rate of Fe was higher than that of Mn because the latter is less soluble than the former, with a slight sulfate reduction to sulfur.

Methane generation has not yet been achieved, but as the redox ladder predicts, methanization of the lake will be a matter of time. Lake formation is a complex process that lasts for long periods of time. In this case, there has been the opportunity to monitor the formation process from the beginning, identifying the different chemical processes that lead to its methanization. Likewise, it has served to verify in which phase of the methanization process the lake is once formed.

## Data Availability

Data not available.

## Declarations

All authors have read, understood, and have complied as applicable with the statement on “Ethical responsibilities of Authors” as found in the Instructions for Authors and are aware that with minor exceptions, no changes can be made to authorship once the paper is submitted.
